# Health, Architecture and Wellbeing: building bridges between health and the design of living spaces

**DOI:** 10.1093/eurpub/ckac129.299

**Published:** 2022-10-25

**Authors:** T Leão, A Neiva

**Affiliations:** 1 Instituto de Saúde Pública da Universidade do Porto, Porto, Portugal; 2 Faculdade de Medicina da Universidade do Porto, Porto, Portugal; 3 Faculdade de Arquitectura da Universidade do Porto, Porto, Portugal

## Abstract

**Background:**

Health is determined by where we live. Nevertheless, medical and architecture students often ignore the relation between health, housing and urban planning. In order to bridge these areas, we designed an interdisciplinary course for students of University of Porto Medical and Architecture schools, winning a 2021 Pedagogic Innovation courses call.

**Objectives:**

”Health, Architecture and Wellbeing” aimed to explore the effect of living spaces in health, combining the health and architecture perspectives in the analysis of case studies, and discussing strategies to improve health and wellbeing of the population. By exposing students to tools for designing healthy, sustainable, efficient and accessible spaces, and to public participation strategies, we aimed to foster awareness and share knowledge among complementary scientific fields.

**Results:**

Students were exposed to theoretical and practical sessions with experts from the medical, architecture, landscape architecture, and geography areas, as well as to field visits in social neighbourhoods, collective housing, and green spaces. Besides introducing students to concepts, as health needs and determinants, and to healthy and accessible design methods, students interacted with inhabitants, gathering experiences on how they lived in those spaces, understanding its impact on their wellbeing, while exploring the enablers and barriers of participation strategies. Students were encouraged to complement technical architecture competences with public health evidence, and social participation leading to the development of design proposals improving the existing living environments.

**Conclusions:**

“Health, Architecture and Wellbeing” interdisciplinary course connected public health expertise with architecture, promoting knowledge dissemination and awareness among health and architecture students. This experience may be reproduced in other countries, as health continues to be determined by living and public spaces.

**Key messages:**

• Bridging public health with other disciplines is fundamental to understand the origin of health issues, namely living conditions, and maximise the opportunities of acting near their root causes.

• This course allowed architecture and medical students to gain awareness about the impact of housing and urban design on people’s health, and to learn how to propose and design healthier environments.

